# Address of the Metropolitan Society of General Practitioners to Their Brethren

**Published:** 1830-10-01

**Authors:** 


### ADDRESS.

*Transcribed from the Book of Laws and Regulations of "the Metropolitan Society of General Practitioners in Medicine, &c. &c.*

"Concordia Res Parvæ Crescunt."

AN association has been established in London, denominated "*The Metropolitan Society of General Practitioners in Medicine and Surgery throughout England and Wales,*" the nature of which is developed by this, its first code of laws, whilst its more general intentions and objects are briefly explained in the following statement.

*Medical Men*, in this country, whose services are dedicated to the practice of their profession through all its extensive ramifications of Medicine, Surgery, Pharmacy and Midwifery, have been aptly denominated "*General Practitioners.*" The epithet, as distinguished from the appellations which designate those individuals who devote themselves to *one branch only* of the healing art, *is as honourable* as it is descriptive; inasmuch as it denotes the possession of qualifications *adequate to all* the emergencies of an arduous profession. It has, however, been said, that, in its relation with the titles of "Physician," and "Surgeon," the term "General Practitioner" implies a *Subordinate* in the social and intellectual ranks of the republic of Medicine; but such an inference is at variance with the spirit of the designation, and presents a forced acceptation of its sense, to which no individual of the class will subscribe. It will hereafter be the duty of the Association now established under the denomination of "*The Metropolitan Society of General Practitioners,*" to discuss the subject; and, after due examination and deliberation, to confirm this or adopt another cognomen. It will, also, be the province of the Society, to institute an inquiry into the expediency of equalizing the right to professional distinctions, and to adopt such policy as shall secure for its Members the civil and literary respect to which their education, attainments, and practice entitle them.

The position in society occupied by General Practitioners, is one that demands their serious attention. Perplexed by multifarious duties—threatened by extensive responsibilities—oppressed by physical exertions—disturbed by conflicting interests—assailed by jealousies—harrassed by intrigue and envy—injured by corporate privileges—insulted by legal enactments, and degraded by an opprobrious mode of remuneration—the General Practitioner has more extensive evils to cope with, than he can hope to combat successfully by the unassisted force of his own mental and physical exertions. It is, therefore, a subject of astonishment, that the members of a class, around whose banner more than ten thousand individuals are spread over the cities and provinces of England and Wales, have not sooner coalesced, and formed themselves into a deliberative body with executive authority and means, in order to render the knowledge, experience, and resources of the entire mass, available to every Member of the Association, who might seek or require its advice and support. By such an union, a concentration of the opinions, experience, talents, and influence of the whole class would be consummated, and its application directed upon all occasions, to the necessities and emergencies of any individual; or to the promotion of the collective interest of the whole body. In aid of a co-operative system like this, the support derived from a pecuniary fund is not to be overlooked; in fact, it is an indispensable requisite for carrying into effect any political or legal undertaking—for defending individual interests—and for supporting a domiciliary establishment, which, to ensure success to the scheme, should offer, not merely a place for the conduct of business, but the conveniences for agreeable social intercourse.

Upon the foregoing principles has the Society of General Practitioners been begun; its prosecution may be understood by the following details.

In the selection of a House for the Society's use, the Committee have been influenced by a prudent regard to economy, and a desire to restrict the extent of the Chambers to the actual necessities of the Association. The Committee did not consider it justifiable in the onset, to open an establishment upon a large scale; purposing to extend it, whenever an increase of the Society's numbers renders it necessary, or the Members themselves may call for further accommodation. Such refreshments as can be prepared under the present circumscribed fitness of the premises, are served (at a moderate charge) at any time during the day, by the persons in attendance. It is in contemplation, however, to provide dinners and other refection, as soon as the magnitude of the Society will warrant the adoption of a plan for combining the comforts and conveniences of a social club, with the more solid advantages to be derived from the Institution. The Reading Room is open from ten o'clock in the morning till ten at night; and the Daily Newspapers, periodical journals, &c. regularly laid on the table. Notwithstanding the Library offers at this time but a limited allurement, the Society has cause for congratulation in the prompt and handsome manner in which many eminent individuals, not belonging to the Association, have presented their works. The Members of the Society, also, have not been tardy in offering their respective donation of books. The Library will be opened as soon as the necessary arrangements are completed.

The Committee have great pleasure in announcing, that the Treasurer's statement of the Society's affairs, shows *a balance of cash in hand;* and they take this opportunity of giving a pledge to its Members and to the profession at large, that they will, on no account, incur any liabilities beyond the actual resources of their funds.

The pleasure and advantages to be derived by the Metropolitan Surgeons, from the social and friendly intercourse established and confirmed through the medium of their Chambers, are too apparent to need any comment: to the *country* Members, also, they present a most convenient place of resort during their occasional sojourn in London, where they may meet their professional friends, mix with their unknown contemporaries, and, at leisure, contemplate the men, books, customs, manners, opinions and feelings, of the medical microcosm of the Metropolis. The Associates, likewise, join in these literary and social meetings; forming and cementing those professional ties and private friendships which ought to subsist between all the members of a liberal profession. To the student in Medicine also, the Society's Chambers afford peculiar advantages. He is supplied with books either of reference or general instruction; he has the accommodation of a comfortable room for meeting his friends, for his moments of leisure and relaxation or literary studies; and lastly, in addition to the mental and scientific improvement which he derives from attending the discussions of the Society, he associates daily with his seniors in the profession, from whose conversation and communications he receives both pleasure and instruction.'

The amount of the annual contribution, in comparison with the advantages to be derived from it, has been fixed at a very moderate sum; the Committee feeling assured that the numbers of the Society will be equal, even at this small ratio, to the production of a fund amply sufficient for all the purposes required. The first care of the Society is the conservation of its own integrity, and the general interests of its Members. Medical politics have decidedly taken a bias, unfavourable to the General Practitioner, and he stands, not only unprotected in his professional character by the fostering hand of a generous government, but legislative enactments have actually been passed, which oppress and degrade him; his privileges are trampled down by the assumptions of unjust, self-created, arbitrary power; and the defence of his rights confounded by the hazardous jurisprudence of legal mis-interpretation. These great and crying evils can only be redressed by parliamentary influence; and the chief strength of the fund arising from the contributions of the Members of this Society, lies in the power which it gives, of appealing to the legislature, and of persisting steadily against oppression and opposition, until the General Practitioner shall have obtained a distinct and legal recognition of his rights, privileges, and rank, and have burst every trammel that binds him down to a degraded subserviency. These are measures which the Society is pledged to pursue; the period of their commencement must, of course, depend upon the possession of means, and be fixed by the *fiat* of deliberation. For the purposes of individual protection, the fund will at all times be available in every instance where, upon due inquiry and examination, it shall appear to the Society, that one of its Members sustains any injury or wrong in his professional capacity; or is called on to assert his right or defend his interest on any point that applies strictly and especially to the whole body. The subject of professional remuneration is of momentous urgency, and demands the most careful consideration. It is true, that, under the direction of the Lord-Chief-Justice Tenterden, a verdict was lately given in favour of the right of a General Practitioner to charge for his services; but such a decision by no means necessarily becomes a law of the land, and though dictated by the opinion of one judge to-day, it may be reversed by the *dictum* of another to-morrow. When the Society shall have arranged a scheme for regulating a general mode of professional compensation, by which the Medical Practitioner may be emancipated from the odious necessity of balancing his remuneration by the charge for his medicines, it will be necessary to legalize the measure by an application to Parliament. In fine, the fund formed by the annual contributions, will enable the Society to prosecute measures for obtaining such legislative interference as may be necessary in removing all disabilities; for the protection and support of the interests and welfare of its Members; for bringing into operation those suggestions which the fluctuating influence of circumstances may give rise to; and for establishing the respectability and prosperity of the General Practitioners of this kingdom.

The plan of the benevolent fund differs from any other heretofore established;* being founded upon the principle of *general* benevolence. To the Members of this Society, whose circumstances preclude them from providing for the contingencies of accident, disease, old age, and death, it must surely be a consolation to contemplate a resource for such periods of desolation; whilst the more favoured individuals whom fortune has placed above the necessity for such aid, will not withhold their support to the efforts of humanity. It is, therefore, confidently trusted, that the voluntary donations of all classes of persons, will not fail to produce a fund adequate to the benevolent intentions of its philanthropic contributors; in aid of which the *surplus of the general fund* will be added to it, as often as it exceeds the sum necessary for the exigencies of the Society.

To promote the objects contemplated by the founders of this Society, its Members, wherever situated, are invited, at all times, to an unreserved communication of their opinions and wishes. By this means a splendid system of general co-operation will be established throughout the kingdom, and remedies devised and applied for evils of every sort, whether local or universal, individual or collective. In furtherance of the measures for improving the science of medicine, it is requested that the Members will transmit to the Society in London, such papers, reports of cases, and other professional information, as they may consider conducive to the improvement of knowledge and the good of the public. The more important communications will be printed, as the "*Transactions*" of the Society; and as the literature thus collected, will be regarded as the joint property of the Association, it will be published for the benefit of its Members, merely at a remunerating price for the expenses incurred.

Such are the principles and intentions of a Society to which all the General Practitioners in the kingdom should attach themselves; and every individual of the class is hereby invoked, by the respect which he bears for himself and the regard he entertains for the honour and interest of his profession, to give his aid in promoting the formation of an institution for establishing the prosperity and happiness of the medical community.

"Ex veritate causa pendetur."

By order of the Committee,
HENRY BOND, Sec.

*Society's Chambers*, 4, *Regent Street*,
*August* 12, 1830.

*It is requested that all applications and communications be addressed (post paid) to the Secretary. For the mode of admitting Members into this Society, vide Chap.* 1, *Clause* 8, *of the Rules and Regulations.*

* Extending relief to Medical men who do *not belong* to the Society, and are *not* subscribers to any fund.

